# Dipeptidyl-peptidase 4 (DPP4) mediates fatty acid uptake inhibition by glucose via TAS1R3 and GLUT-2 in Caco-2 enterocytes

**DOI:** 10.1016/j.heliyon.2024.e30329

**Published:** 2024-04-25

**Authors:** Verena Preinfalk, Isabella Kimmeswenger, Veronika Somoza, Barbara Lieder

**Affiliations:** a Christian Doppler Laboratory for Taste Research, Institute of Physiological Chemistry, Faculty of Chemistry, University of Vienna, Vienna, Austria; b Vienna Doctoral School in Chemistry (DoSChem), University of Vienna, Vienna, Austria; c Institute of Physiological Chemistry, Faculty of Chemistry, University of Vienna, Vienna, Austria; d Leibniz Institute for Food Systems Biology at the Technical University of Munich, Freising, Germany; e Institute of Clinical Nutrition, Department of Human Nutrition and Dietetics, University of Hohenheim, Stuttgart, Germany

## Abstract

Both high glucose intake with a high-fat meal and inhibition of dipeptidyl peptidase-4 (DPP4) have been associated with plasma lipid-lowering effects, but mechanistic understanding linking glucose and fat absorption is lacking. We here hypothesized that glucose ameliorates intestinal fatty acid uptake via a pathway involving DPP4. A concentration of 50 mM glucose reduced mean DPP4 activity in differentiated Caco-2 enterocytes by 42.5 % and fatty acid uptake by 66.0 % via nutrient sensing by the sweet taste receptor subunit TAS1R3 and glucose transporter GLUT-2. No effect of the DPP4 substrates GLP-1 and GIP or of the cellular energy status on the reduced uptake of fatty acids was seen, but a direct interaction between DPP4 and fatty acid transporters is suggested. Conclusively we identified DPP4 as a regulator of fatty acid absorption in Caco-2 enterocytes that mediates the inhibition of intestinal fatty acid uptake by glucose via an interplay of GLUT-2 and TAS1R3.

## 1 Introduction

Dipeptidyl-peptidasse-4 (DPP4), also known as CD26, is a membrane-bound peptidase. It is ubiquitously expressed on the surface of many cell types and is also found in the bloodstream, highlighting DPP4′s dual role as a membrane-bound and soluble isoform [[1], [2], [3]]. The cell-surface aminopeptidase DPP4 has a multifunctional serine protease activity that is involved in metabolizing substrates and has been implicated in several metabolic disorders including non-alcoholic fatty liver disease, or as several experts now call it, metabolic dysfunction-associated steatotic liver disease (MASLD) [4], cardiovascular disease, and obesity [[5], [6], [7], [8]]. However, DPP4 is more common as a key player in regulating glucose homeostasis and anorexigenic signaling through GLP-1 and GIP degradation [9,10]. Thus, inhibiting DPP4 activity is an effective strategy for reducing blood glucose levels by preventing the breakdown of plasma GLP-1 for the treatment of type 2 diabetes mellitus (T2DM) [11]. Besides the effect on blood glucose, it was previously described that inhibition of DPP4 activity has positive effects on lipid metabolism. DPP4 inhibitors like vildagliptin [12] and sitagliptin [13] suppressed postprandial elevation of triglycerides and ameliorated dyslipidemia in T2DM patients [14]. Furthermore, these inhibitors have been shown to elicit a lipid-lowering effect in meta-analyses [14,15]. These findings indicate that DPP4 inhibition reduces plasma lipid levels, although the underlying mechanism is not yet understood.

DPP4 has a minor effect on gastric emptying, thus a possible reduction of intestinal fatty acid uptake as a mechanism for delayed postprandial increase of triglycerides has been suggested [16]. However, mechanistic approaches to substantiate a direct action of DPP4 on intestinal fatty acid uptake is lacking in the literature. Intestinal fatty acid uptake in enterocytes is mediated via a passive diffusion mechanism and a protein-mediated mechanism [17]. The latter is described as the primary mechanism, where fatty acids are taken up by fatty acid transporters on the apical membrane of enterocytes [18]. A larger number of fatty acid transporters has been identified, including fatty acid translocase 36 (CD36), plasma membrane-associated fatty acid-binding protein (FABP), and fatty acid transport proteins (FATP) 1–6. FATP 4, FABP, and CD36 are discussed to be the most relevant transporters in enterocytes [17].

Fatty acid transporters are also regarded as a promising therapeutic target to regulate lipid uptake in an organ-specific manner [19] and a close connection to carbohydrate metabolism exits via insulin. Insulin not only facilitates cellular glucose uptake but is also a major stimulus for fatty acid uptake via translocation of CD36 and other fatty acid transporters [20]. This outlines the importance of an improved understanding of the interaction between glucose and lipid metabolism. Habitual daily meals are composed of a mixture of foods that provide a variety of nutrients, including lipids, as well as digestible and indigestible carbohydrates. That said, potential interactions of nutrients should be considered. Since Western diets are high in fat and digestible carbohydrates [21], more knowledge is required about the interactions between carbohydrate and lipid metabolism, which might also play a role in metabolic health conditions such as diabetes mellitus considering DPP4 as a potential link.

It has been demonstrated that the presence of carbohydrates in the intestine affects the absorption and metabolism of lipids [22]. The addition of 50 g and 100 g glucose to fatty test meals decreased postprandial triglyceridemia in healthy adults [23]. However, the direct impact of glucose on intestinal fatty acid uptake has not been addressed on a mechanistic level. To address this research gap, we aimed to study the impact of glucose on fatty acid uptake in enterocytes and propose a regulation of the DPP4 activity as a mechanistic link. We hypothesized that glucose ameliorates intestinal fatty acid uptake via a pathway involving DPP4. Since there is evidence that both the sweet taste receptor and glucose transporters are important for the intestinal nutrient sensing of carbohydrates [24,25], we investigated the role of glucose transporters and sweet taste receptors in the underlying signaling pathway. For this purpose, Caco-2 cells were used as a model which resembles intestinal enterocytes upon differentiation [26], and fatty acid uptake and DPP4 activity were studied after treatment with the monosaccharide glucose. The pathway analysis focused on the role of nutrient sensing via the sweet taste receptor subunit TAS1R3 and glucose transporters, while excluding an effect on passive diffusion and barrier integrity, the requirement of metabolization of glucose, and a role for the DPP4 substrates GLP-1 and GIP.

## 2 Methods

### 2.1 Materials

All chemicals and reagents were purchased from Sigma-Aldrich (Austria), Carl Roth (Austria), Cayman Chemical Company (Austria) or Thermo Fisher Scientific (Austria) unless stated otherwise. The human colon carcinoma cell line Caco-2 was obtained from CLS Cell Line Services (Germany).

### 2.2 Cell culture

Caco-2 cells were used as a model for intestinal enterocytes as described previously [[26], [27], [28]]. Upon differentiation for 21 days, Caco-2 cells develop enterocyte-specific characteristics and have been described as a suitable model for the intestinal brush border membrane [26,29]. Caco-2 cells were cultured in Dulbecco's modified Eagle's medium (DMEM) supplemented with 10 % fetal bovine serum (FBS), 2 % stable L-glutamine (glutamax®), and 1 % penicillin/streptomycin at 37 °C and 5 % CO_2_ at humidified atmosphere. Cells were passaged at 80–90 % confluence and used until passage 25.

The cultivation of the cells was carried out in the same way for all experiments. Cells were seeded in appropriate plate formats at a density of 2 × 10^5^ cells per cm^2^ and the medium was changed every second to third day until the fully differentiated cells were used for experiments on day 21 ± 3. The formation of an intact monolayer for uptake studies was assured by a TEER value of at least 330 Ω x cm^2^ [30,31].

### 2.3 Cell viability

Negative effects of all test compounds on metabolic activity were excluded via MTT (3-(4,5-dimethylthiazol-2-yl)-2,5-diphenyltetrazolium bromide) assay. Cells were treated according to the conditions used for the fatty acid uptake as described below. Briefly, Caco-2 cells were differentiated in 96-well plates and starved for 60 min in serum-free medium before incubation with the test compounds dissolved in HBSS/HEPES for 30 min at 37 °C. The medium with the test compounds was replaced by the MTT working solution, containing a final concentration of 0.83 mg/mL of MTT dissolved in phosphate-buffered saline (PBS). After incubating the cells for 5–10 min, the MTT working solution was removed and DMSO was added to dissolve the formed purple formazan salt. Absorbance was measured at 550 nm with 690 nm as a reference wavelength using a multimode plate reader (Spark, Tecan, Switzerland). The metabolic activity of treated cells was assessed relative to untreated control cells.

### 2.4 Fatty acid uptake

Free fatty acid uptake in differentiated Caco-2 cells was determined by QBT™ fatty acid uptake kit (Molecular Devices Germany GmbH, Germany) as described previously [[26], [27], [28]]. The assay is based on the trafficking of fluorescently labeled fatty acid analogue BODIPY-C_12_ into the cells, resulting in intracellular fluorescence at the same time. Caco-2 cells were differentiated in 96-well plates for 21 ± 3 days and starved for 60 min in serum-free medium before adding the test compounds dissolved in HBSS/HEPES. The loading dye containing BODIPY-C_12_, diluted in HBSS/HEPES with 0.1 % (w/v) essential fatty acid free bovine serum albumin (BSA), was added after 30 min pre-incubation with the test compounds. The fluorescence signal emitted upon uptake of the BODIPY-C_12_ was recorded at 515 nm emission and 485 nm excitation every 20 s for 60 min using a multimode plate reader (FlexStation III, Molecular Devices, Germany). The area under the curve (AUC) resulting from the respective signal/time plots was used to calculate the mean fatty acid uptake over time relative to the corresponding control treated cells.

### 2.5 Analysis of passive diffusion and monolayer integrity

To evaluate passive diffusion along the Caco-2 monolayer, lucifer yellow permeability assay and transelectrical epithelial resistance (TEER) measurements were performed. In addition, the amount of glucose transported was determined by measuring glucose concentrations at the basal and apical sides of the enterocyte model.

Caco-2 cells were cultured in 24-transwell inserts with a pore size of 0.4 μm (Sarstedt, Germany). After 21 ± 3 days, the cells were treated according to the fatty acid uptake measurements, the cells were starved for 60 min with serum-free medium before incubation with the test compounds dissolved in HBSS/HEPES for 30 min at 37 °C. The TEER value was measured using the EVOM resistance meter (World Precision Instrument, Germany) in combination with a chopstick electrode (World Precision Instrument, Germany) every 15 min over a period of 90 min. Resistance was normalized to the initial resistance prior to test compound addition and the data are presented as the corresponding unit area resistance (Ω x cm^2^).

For the lucifer yellow assay, cells were starved for 60 min with serum-free medium and subsequently incubated with lucifer yellow (LY) solution (100 μM) in combination with the compounds of interest at 150 rpm on a plate shaker at 37 °C for 120 min. Samples of the basolateral compartment were collected after 15, 30, 60, 90, and 120 min of incubation with LY at the apical compartment. The fluorescence signal of the samples was measured in duplicates on a plate reader (Tecan Infinite M200, Tecan, Maennedorf, Switzerland) at 428 nm excitation and 536 nm emission and the amount of LY in the basal compartment was calculated using an external calibration curve of LY.

Glucose transport from the apical to the basolateral side was analyzed after the differentiation of Caco-2 cells in transwell inserts (Sarstedt, Germany). The cells were treated according to the conditions used for fatty acid uptake analysis as described above and medium samples were taken from the apical and basal compartments, resembling the apical and basolateral side. The glucose concentration in each compartment was determined using a colorimetric assay (Glucose Colorimetric Assay Kit, Cayman Europe, Estonia) according to the manufacturer's protocol with a plate reader at 514 nm. Glucose concentrations were calculated using an external standard curve and compared to the control treatment.

### 2.6 Analysis of reactive oxygen species formation

Cellular reactive oxygen species (ROS) were measured using the DCFDA/H_2_DCFDA – cellular ROS assay kit (Abcam, United Kingdom) according to the manufacturer's protocol. For the assay, cells were seeded in 96-well plates and differentiated for 21 ± 3 days. On the day of the experiment, cells were starved for 60 min in serum-free medium before being incubated with the test compounds for 30 min at 37 °C, and stained with 50 mM DCFDA solution for 45 min at 37 °C in the dark. Carbonyl cyanide-p-trifluoromethoxyphenylhydrazone (FCCP, 5 μM), oligomycin (0.5 μM) and *tert*-butylhydroperoxid (TBHP, 200 μM) were used as positive controls. ROS generation was evaluated after 90 min with a fluorescent microplate reader (Flex Station III, Molecular Devices, Germany) at an excitation wavelength of 485 nm and an emission wavelength of 535 nm. Fold changes from the assay controls and glucose treatments were calculated as a percentage of the control treatment.

### 2.7 DPP4 enzyme activity

Differentiated Caco-2 cells, cultured in 12-well plates, were starved with serum-free medium for 60 min and further treated according to the conditions used for the fatty acid uptake with 10 or 50 mM glucose with or without the addition of 250 μM phloretin as a GLUT-2 inhibitor or 1 mM lactisole as a TAS1R3-inhibitor for 30 min at 37 °C. After a washing step with PBS, the cells were lysed with 10 mM Tris-HCl +0.1 % (w/v) BSA and subsequently centrifuged for 10 min at 16,000×*g* and 4 °C. The cellular DPP4 enzyme activity in the cell lysate was determined utilizing a commercial DPP4 Protease Assay (Promega GmbH, Walldorf, Germany). The signal in relative light units (RLU) which is proportional to DPP4 activity was measured within 30 min using a multimode plate reader (Flex Station III, Molecular Devices, Germany). The obtained RLU signal was normalized to the total protein concentration of the lysate, which was determined by the Bradford assay [32]. Data is shown as the difference to the control treated cells in %.

### 2.8 GIP and GLP-1 release in differentiated Caco-2 cells

Caco-2 cells were cultured in 12-well plates and starved for 60 min with serum-free medium prior to the incubation with the test compounds for 30 min at 37 °C. The cell monolayer was scraped from the bottom of the wells to transfer the supernatant and cells into a reaction tube containing a commercial DPP4 inhibitor (Sigma-Aldrich) to prevent degradation. The cells were lysed by repeated aspiration through a 20G cannula. The reaction tubes were then placed on ice and gently shaken for 1 h. GLP-1 and GIP concentrations were determined using commercially available ELISA kits (GLP-1 total ELISA Kit, EMD Millipore Corporation and GIP Enzyme Immunoassay Kit, RayBiotech) according to the manufacturer's protocol using a multimode plate reader (Spark, Tecan, Switzerland) at a wavelength of 450 nm and 590 nm as a reference wavelength. GLP-1 and GIP concentrations were calculated with an external standard curve and normalized to the total protein content determined by the Bradford assay [32]. Data are shown relative to the control treatment.

### 2.9 Quantitative real-time polymerase chain reaction

To confirm the expression of selected genes of interest involved in fatty acid uptake regulation, RT-qPCR experiments were performed. Fully differentiated Caco-2 cells were starved for 60 min with serum-free medium followed by incubation with the test compounds for 30, 60 and 90 min at 37 °C. Afterwards, RNA was isolated using Monarch® Total RNA Miniprep Kit (New England Biolabs) and reverse-transcribed to cDNA using LunaScript® RT SuperMix Kit (New England Biolabs) according to the manufacturer's protocol. PCR was subsequently performed using Luna® Universal qRT-PCR Master Mix (New England Biolabs) on a Step-One Plus Device (Applied Biosystems, Thermo Fisher Scientific, Austria). The primer pairs used during the PCR can be found in Table S1. Primers not previously reported, namely for *DPP4*, *GCG*, *GLP1R*, *GIP* and *GIP-R*, were validated by sequencing the obtained PCR product (Sanger Sequencing, Eurofins, Germany). Gene expression is given as fold change compared to the corresponding control. The starting concentrations of the respective mRNA used for reverse transcription were calculated using LinRegPCR Version 2021.2 and compared to the corresponding control after normalization to *HPRT1* and *GAPDH* as reference genes.

### 2.10 Statistical analysis

In vitro data are displayed as means ± SEM, unless stated otherwise, from at least three biological and two technical replicates. To exclude outliers from statistical analysis, the Nalimov outlier test was applied. The data sets were controlled for normal distribution and equal variances. To test the significant difference between test compounds versus corresponding control cells, a one-way analysis of variance (ANOVA) followed by Tukey's multiple comparison test or Dunnett's multiple comparison test (as described in the figure legends) was performed. Two-way ANOVA was carried out to analyze differences between treatments and time. Statistical analysis was performed using GraphPad Prism 9.4.0 or higher.

## 3 Results

### 3.1 Cell viability

MTT assays were applied to exclude negative effects on cell viability after the treatment of Caco-2 cells with the test compounds in the applied concentrations and incubation times. No significant differences (p > 0.05) were detected between treatments and untreated control cells (data not shown).

### 3.2 Glucose dose-dependently inhibits fatty acid uptake in Caco-2 cells

**Fig. 1:**
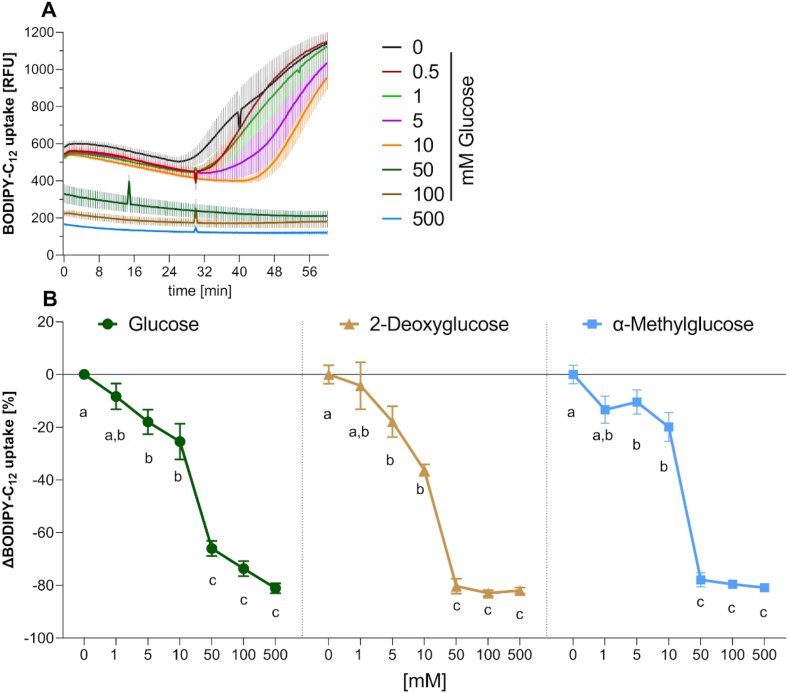
**BODIPY-C**_**12**_**uptake by differentiated Caco-2 cells. A)** Mean kinetic measurements of BODIPY-C_12_ uptake over 60 min in relative fluorescent units (RFU). n = 3 (tr = 2) ± SEM. **B)** BODIPY-C_12_ uptake after treatment with Glucose, 2-Deoxyglucose and α-Methylglucose. Data are presented as Δ means compared to corresponding control ± SEM n = 3 (tr = 2). Significant differences were tested using two-way ANOVA with Tukey's multiple comparisons test. Different letters indicate significant differences within different treatments compared to control treatment (p < 0.05).

To test the impact of a glucose load on intestinal fatty acid uptake, we treated Caco-2 cells with different glucose concentrations in the range of 0.5–500 mM. Fig. 1A shows the mean of the kinetic readings of fatty acid uptake in Caco-2 cells in response to the treatment with different glucose concentrations. Under control conditions, a rising relative fluorescence unit (RFU) signal at around 30 min was detected, indicating an increasing uptake of fatty acids by the differentiated Caco-2 cells. The RFU signal was decreased in response to the glucose treatment in a dose-dependent manner. The fatty acid uptake over a time span of 60 min is depicted in Fig. 1B as the area under the curve (AUC) compared to the control. Incubation with 5–500 mM glucose dose-dependently reduced fatty acid uptake over time in a range from −17.98 ± 11.5 % (5 mM glucose; p < 0.05) up to −81.11 ± 4.6 % (500 mM glucose; p < 0.0001) compared to control treated cells.

### 3.3 The inhibitory effect of glucose on fatty acid uptake is independent of the metabolization of glucose

Glucose serves as the primary substrate for glycolysis [33], we thus examined whether glycolytic breakdown of glucose is required for the fatty acid uptake inhibition. Caco-2 cells were incubated with 2-deoxyglucose (2-DG), which is absorbed via GLUT-2, but not further metabolized, in concentrations ranging from 1 to 500 mM. As with glucose, the uptake of fatty acids declined with increasing 2-DG concentration (p < 0.0001) (Fig. 1B). A similar dose-dependent reduction of fatty acid uptake was obtained after incubation with α-methyl glucose, which is absorbed via the SGLT-1 but also not further metabolized by glycolytic pathways (p < 0.01; Fig. 1B). The effect size of 2-DG and α-methyl glucose was not different from that of glucose (p < 0.05), which argues against the metabolization of glucose being essential for the effect of glucose on fatty acid uptake. Notably, a substantial leap in the effect size between 10 and 50 mM glucose or the glucose analogues was observed (p < 0.0001). These two concentrations were selected for the mechanistic analysis of glucose's effect on fatty acid uptake, as we hypothesized a role for the translocation of GLUT-2 to the apical side at higher glucose concentrations.

Since cellular glucose metabolism is associated with increased production of reactive oxygen species (ROS) [34,35], we further investigated whether cellular ROS production induced by high glucose concentrations affects fatty acid uptake. The results show that the tested glucose concentrations of 10 and 50 mM had no significant impact (p > 0.05) on ROS production (Fig. S1).

**Fig. 2:**
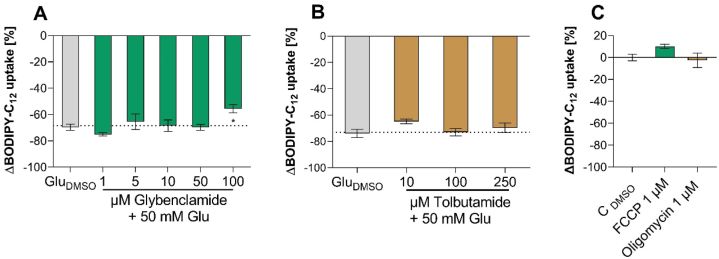
**BODIPY-C**_**12**_**uptake by differentiated Caco-2 cells** after treatment with **A)** Tolbutamide, and **B)** Glybenclamide. **C)** the mitochondrial uncoupler FCCP and oligomycin. Data are presented as Δ means compared to corresponding glucose with DMSO control ± SEM n = 3–4 (tr = 2). Significant differences were tested using one-way ANOVA with Dunnett's multiple comparisons test. Asterisk indicates a statistically significant difference (p < 0.05).

We further confirmed that the metabolization of glucose is not essential for its effects on fatty acid uptake in experiments regarding the impact of the energy status of the cell. First, the energy sensor ATP-sensitive potassium channel (K_ATP_), which was also described as an important component of the STR-independent sweet taste signaling pathway [[36], [37], [38]], was investigated. Two different K_ATP_ channel blockers, glybenclamide and tolbutamide, were tested. Co-incubation with the highest concentration of glybenclamide (100 μM) resulted in a slightly higher fatty acid uptake (p < 0.05), compared to the glucose treatment (Fig. 2A). However, this was not confirmed by co-incubation of the cells with 10–250 μM tolbutamide and 50 mM glucose (Fig. 2B).

The ATP status of the cell as a mediator of glucose-induced fatty acid uptake inhibition was further investigated via induction of mitochondrial uncoupling. We incubated Caco-2 cells with FCCP (carbonyl cyanide p-trifluoromethoxyphenylhydrazone) and oligomycin. FCCP is an uncoupler of oxidative phosphorylation in mitochondria that disrupts ATP synthesis [39] and oligomycin inhibits H^+^-ATP-synthase [40]. Neither FCCP nor oligomycin had a significant effect (p > 0.05) on the fatty acid uptake in Caco-2 cells (Fig. 2C).

Taken together, these results demonstrate that neither the metabolization of glucose nor the ROS production or the ATP status of the cell is mediating the inhibitory effect of glucose on fatty acid uptake in Caco-2 cells.

### 3.4 Glucose does not change passive diffusion of fatty acid uptake

**Fig. 3:**
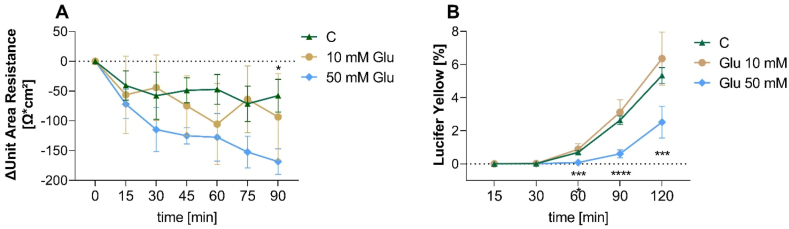
**Effects of glucose treatments in differentiated Caco-2 cells. A)** Evaluation of the *trans*-epithelial electrical resistance (TEER) after treatment with 10 or 50 mM glucose for 0, 15, 30, 45, 60, 75, and 90 min or non-treated control cells. Values are presented as Δ means ± SEM for three experiments (tr = 2). **B)** Evaluation of permeability of lucifer yellow (LY) in % after treatment with 10 or 50 mM glucose or non-treated control cells for 15, 30, 60, 90, and 120 min. Data are presented as means ± SEM for three experiments (tr = 2). Statistically significant differences were analyzed using two-way ANOVA with Holm-Sidak post-hoc test. Asterisks indicate statistically significant differences compared to control (C) (*p < 0.05, ***p < 0.001, ****p < 0.0001). (For interpretation of the references to color in this figure legend, the reader is referred to the Web version of this article.)

Next, we assessed whether glucose treatment affects barrier permeability. For this purpose, TEER values were monitored over 90 min after incubation with 10 or 50 mM glucose. A significantly lower TEER value (p < 0.05) was shown after 90 min for the 50 mM glucose treatment compared to the control treatment (Fig. 3A). However, the results from the analysis of the barrier integrity by monitoring the permeability of the cell monolayer for the fluorescent molecule lucifer yellow do not support that this TEER decrease increased the permeability of the cell membrane. In contrast to the TEER results, the higher glucose concentration of 50 mM led to a reduced concentration of lucifer yellow compared to the control treatment (p < 0.001, Fig. 3B) starting after 60 min, indicating a reduced permeability after 50 mM glucose exposure. Glucose transport from the apical to the basolateral side was not affected as there was no shift in glucose concentrations after 90 min at the apical and the basolateral side after the treatment with 10 or 50 mM (p > 0.05, data not shown in the figure).

### 3.5 Effect of glucose on gene expression in Caco-2 cells

**Fig. 4:**
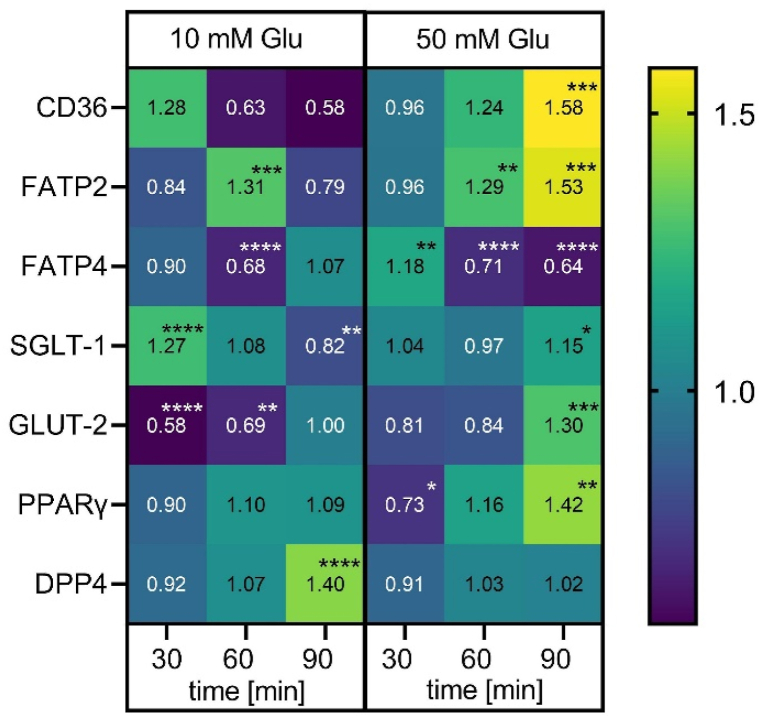
**qPCR analysis of marker genes for glucose and fatty acid absorption.** Heatmap representing the mean fold change (n = 3–4, tr = 3) after treatment with 10 or 50 mM glucose for 30, 60, and 90 min compared to the control treatment. Statistical significance was analyzed using two-way ANOVA with Holm-Sidak post-hoc test. n = 3–4 (tr = 3), asterisks indicate statistically significant differences compared to control (*p < 0.05, **p < 0.01 ***p < 0.001, ****p < 0.0001).

To evaluate the effect of glucose treatment on gene expression of different proteins related to cellular glucose and fatty acid uptake and metabolism, RT-qPCR analysis was performed. As shown in Fig. 4, a regulation of the gene expression level was demonstrated for several genes of interest. Glucose concentrations of 10 and 50 mM differentially altered gene expression of the target genes. The relative expression for *CD36* declined with increasing incubation time if treated with 10 mM glucose, whereas the treatment with 50 mM glucose led to a 1.74 ± 0.19 times higher fold change compared to control treated cells (p < 0.001). The gene encoding for FATP2 was upregulated after 90 min incubation with glucose (p < 0.001) similar to *PPARG* (p < 0.01), whereas the expression analyses for the gene targeting FATP4 showed a decreased expression (p < 0.0001). The variations in gene expression of *CD36*, *SLC27A2* and *DPP4* were in the range of the control treatment after 30 min incubation with glucose. Treating Caco-2 cells with 10 mM, but not 50 mM glucose for 90 min resulted in an increased *DPP4* gene expression (p < 0.0001). For the target gene encoding for GLUT-2, a significant downregulation occurred already after a 30 min incubation with 10 mM glucose (p < 0.0001), whereas the incubation with 50 mM glucose resulted in a slight upregulation to 1.30 ± 0.08 (p < 0.001) after 90 min.

### 3.6 Glucose inhibits DPP4 activity in Caco-2 cells and DPP4 activity is required for fatty acid uptake

**Fig. 5:**
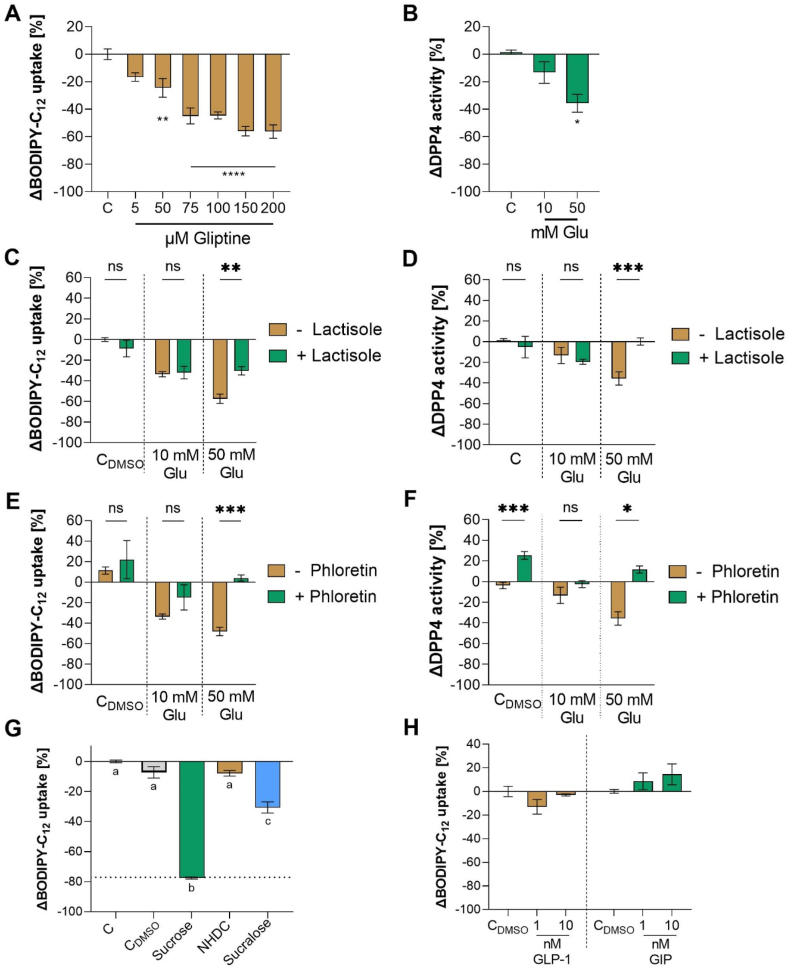
**BODIPY-C**_**12**_**uptake by differentiated Caco-2 cells** after **A)** treatment with a commercially available DPP4 inhibitor mix of gliptines **C)** co-incubation of 1 mM lactisole with 10 or 50 mM glucose, **E)** co-incubation of 250 μM phloretin with 10 or 50 mM glucose, **G)** treatment with sucrose (150 mM), neohesperidin-dihydrochalcon (NHDC, 0.1 mM) and sucralose (0.2 mM) in equi-sweet concentrations corresponding to 5 % sucrose-equivalents, and **H)** incubation with GLP-1 and GIP 1 compared to control. **DPP4 activity in differentiated Caco-2 cells** after **B)** treatment with 10 or 50 mM glucose **D)** co-incubation of 1 mM lactisole with 10 or 50 mM glucose, and **E)** co-incubation of 250 μM phloretin with 10 or 50 mM glucose. Data are presented as Δ means compared to corresponding control ± SEM n = 3 (tr = 2). Significant differences were analyzed using one-way ANOVA with Tukey's or Dunnett's multiple comparisons test. Asterisks indicate statistically significant difference compared to control (*p < 0.05, **p < 0.01, ***p < 0.001, ****p < 0.0001).

Since incubation with higher glucose concentrations is known to affect DPP4 activity in HepG2 cells [41], we hypothesized a similar effect of glucose in Caco-2 cells. Treatment of Caco-2 cells with 50 mM glucose reduced DPP4 enzyme activity by 42.54 ± 2.3 % compared to control (p < 0.05, Fig. 5B). We next tested whether the inhibition of DPP4 activity affects fatty acid uptake. A commercially available DPP4-inhibitor mix of gliptines (Sigma-Aldrich), tested in concentrations from 5 to 200 μM, led to a dose-dependent reduction of fatty acid uptake to a similar extent as glucose (Fig. 5A).

### 3.7 The effect of glucose on DPP4 activity and fatty acid uptake depends on nutrient-sensing by the sweet taste receptor subunit TAS1R3 and glucose transporters

In order to investigate the cellular mechanism behind the reduced fatty acid uptake after glucose treatment, different signal pathways were investigated. Since there is evidence for a role of the sweet taste receptor subunit TAS1R3 as well as for glucose transporters in intestinal nutrient sensing of carbohydrates [24,25], we hypothesized that TAS1R3 mediates the glucose-induced reduction of fatty acid uptake. The involvement of the sweet taste receptor subunit TAS1R3 was assessed using lactisole as an inhibitor [42]. Incubation with 50 mM glucose decreased the fatty acid uptake and DPP4 enzyme activity by −54.16 ± 4.47 % and −42.54 ± 2.3 %, respectively, whereas the concomitant application of 1 mM lactisole reduced this effect (Fig. 5C and D).

To investigate the involvement of glucose transporters, phloretin was used to inhibit uptake via GLUT-2 [43], and the flavonoid phloridzin was used to inhibit SGLT-1 [44]. The co-incubation of 250 μM phloretin and 50 mM glucose resulted in a higher enzyme activity compared to glucose alone (14.40 ± 6.7 %, p < 0.001; Fig. 5E). In contrast, treatment with phloretin solely increased the DPP4 enzyme activity by 27.00 ± 6.9 % (p < 0.05; Fig. 5F). On the other hand, the co-incubation of phlorizin with 10 or 50 mM glucose had no effect (p > 0.05) on the reduced fatty acid uptake provoked by glucose control treatment (data not shown in a figure). No effect was observed for lactisole as well as phloretin after the treatment in combination with 10 mM glucose (p > 0.05), which may be due to the lower effect size of glucose (Fig. 5C–F).

To validate the glucose transporter involvement, we tested the effect of 50 mM sucrose with or without inhibition of α-glucosidase activity, which should prevent the cleavage of the disaccharide sucrose to fructose and glucose.

Like the glucose treatments, the sucrose treatment reduced fatty acid uptake by 77.48 ± 6.1 % compared to the control. Pre-incubation with 10 μM of the α-glucosidase inhibitor voglibose [45] ameliorated the effect of 50 mM sucrose on fatty acid uptake by 25.10 ± 10.2 % (p < 0.05, Fig. S2). The non-caloric TAS1R3 ligands neohesperidin dihydrochalcone (NHDC) and sucralose did not induce an equally strong effect on fatty acid uptake when applied in a similar sweetness range as sucrose (Fig. 5G). This further strengthens the result that glucose transporter activation is required for the strong effect of carbohydrates on fatty acid uptake.

### 3.8 GLP-1 and GIP do not mediate fatty acid uptake inhibition by glucose in Caco-2 cells

Since DPP4 is known to deactivate the incretin hormones glucagon-like peptide 1 (GLP-1) and glucose-dependent inhibitory polypeptide (GIP), we hypothesized that increased concentrations of GLP-1 and GIP may mediate the effect of glucose on fatty acid uptake. First, we analyzed the presence of these incretin hormones in Caco-2 cells. The genes for the GLP-1 receptor as well as GLP-1 were not detectable in differentiated Caco-2 cells. GLP-1 protein expression was at the limit of quantitation of the ELISA (4.1 pM) and was also not stimulated by glucose treatment (data not shown in a figure). In contrast, genes encoding for the GIP receptor and GIP were detectable with qPCR (data not shown in a figure). GIP was detected using a GIP ELISA and the release was significantly enhanced in Caco-2 cells after incubation with 50 mM glucose (p = 0.004, Fig. S3). The incubation with 1–10 nM GLP-1 and GIP solely had no significant effect on the fatty acid uptake compared to control (p > 0.05, Fig. 5H), which excludes a direct effect of the two incretins on fatty acid uptake by differentiated Caco-2 cells.

## 4 Discussion

We here investigated the hypothesis that glucose ameliorates intestinal fatty acid uptake via a pathway involving DPP4, connecting glucose and lipid metabolism.

First, we showed that glucose dose-dependently reduced fatty acid uptake in the enterocyte cell model up to −81.11 ± 4.6 % at the highest test concentrations of 500 mM, which corresponds to sugar concentrations found in soft drinks. We excluded that glycolytic metabolization is required for the effect by applying the non-metabolizable glucose-analogues 2-deoxyglcuose and α-methylglucose. The similar reduction by both glucose-analogues compared to glucose confirmed that the breakdown of glucose via glycolysis is not required for the effect of glucose on fatty acid uptake. Blocking of the cellular energy sensors K_ATP_ channel with glybenclamide had a small impact on the glucose effect on fatty acid uptake (−15.78 ± 11.5 %), but this result was not confirmed with tolbutamide. Thus, no major impact on the ATP-status of the cell can be concluded. A previous study by Ibrahim et al. found that not the glycolytic ATP production, but a mitochondrial uncoupling lead to reduced fatty acid uptake in endothelial cells [46]. However, our results do not support a similar mechanism in Caco-2 enterocytes as none of the two mitochondrial uncoupling compounds tested, FCCP and oligomycin, reduced fatty acid uptake, suggesting that mitochondrial uncoupling is not involved. As glucose in a concentration of 50 mM is described as an inducer of ROS [47], a possible involvement of increased ROS was also considered. However, the incubation with 50 mM glucose did not show any effect on ROS production, concluding that this is not the trigger for the effect of glucose on the reduced fatty acid uptake. Taken together, these results do not point to the involvement of the energy level, they rather suggest a direct impact on fatty acid transporters.

Gene expression analysis of genes encoding for proteins involved in fatty acid absorption showed a stronger down-regulation of the gene encoding for FATP4, a carrier protein for intestinal resorption of long-chain fatty acids [48]. The 10 mM glucose concentration incubated for 60 min also led to a significant reduction compared to control, whereas after 90 min the gene expression was similar to the control level. The reason could be that after the longer treatment time, the lower glucose amounts are already metabolized and not available for stimulation of GLUT-2 and TAS1R3.

In contrast, for the gene targeting FATP2, an upregulation was detected. However, FATP2 compared to FATP4 is considered to be less important regarding intestinal fatty acid absorption [49].

CD36 fulfills important regulatory functions, whereas the transportation of fatty acids is secondary [50]. For *CD36*, the incubation with 10 or 50 mM glucose resulted in opposing effects. Treating Caco-2 cells with 10 mM glucose decreased the gene expression of *CD36*, whereas the 50 mM glucose concentration led to an upregulation of the gene with increasing incubation time. Shiarki et al. observed that an upregulation of CD36 protein in the cardiac muscle resulted in reduced fatty acid serum levels [51]. The elevated gene expression of *CD36* was a result of binding liraglutide. These findings could indicate that GLP-1, stimulated by glucose, binds to *CD36,* leading to an upregulation of the expression, which will result in reduced fatty acid uptake. In this study, the interaction with GLP-1 as a trigger for the affected gene expression can be excluded, as no GLP-1 stimulation in Caco-2 cells could be observed after glucose treatment.

The fatty acid transporter FAT/CD36 is upregulated in the plasma membrane of diabetic mice, which could be prevented by treatment with the DPP4 inhibitor sitagliptin [52]. Our results demonstrate an upregulation of CD36 gene expression in differentiated Caco-2 cells after treatment with 50 mM glucose for 90 min, confirming a connection between high glucose conditions and CD36 regulation. In addition, the DPP4 inhibitors vildagliptin [12] and sitagliptin [13] suppressed postprandial elevation of triglycerides and also ameliorated dyslipidemia in T2DM patients [14]. We, therefore, hypothesized that DPP4 is involved in the inhibitory effect of intestinal glucose on fatty acid uptake. A commercial DPP4 inhibitor, a mix of gliptins, dose-dependently reduced fatty acid uptake, supporting our hypothesis. Ning Gu et al. reported a reduced DPP4 activity in Caco-2 cells after cultivation with 16 mM glucose over 14 days compared to cultivation with 2.5 mM glucose [53]. We here found a reduced DPP4 activity after short-term treatment with 50 mM glucose, but not after exposure to the lower tested concentration of 10 mM, confirming the results by Ning Gu et al. that high glucose conditions are required for a reduction of DPP4 activity. Gene expression analysis of *DPP4* showed significantly higher gene expression after 90 min incubation with 10 mM, but not with 50 mM glucose. This demonstrates that only the enzyme activity is affected by 50 mM glucose treatment, but not the DPP4 gene expression.

Next, we investigated the underlying mechanisms. Nutrient sensing of monosaccharides such as glucose in the intestine is mediated via glucose transporters and the sweet taste receptor [24,25]. We investigated here if the nutrient sensing pathways may also regulate glucose-induced inhibition of fatty acid uptake by applying inhibitors for SGLT-1, GLUT-2, and the sweet taste receptor subunit TAS1R3. Concentrations of 10 mM and 50 mM of glucose were chosen, as it is known that concentrations of 20 mM glucose or higher trigger GLUT-2 migration to the apical side of enterocytes [54]. The inhibition of TAS1R3 and GLUT-2, but not SGLT-1, diminished the effect of 50 mM glucose on fatty acid uptake and DPP4 activity. These results indicate that the nutrient sensing of glucose via TAS1R3 and GLUT-2 plays a stronger regulatory role in fatty acid uptake than the uncoupled ATP phosphorylation in Caco-2 enterocytes, warranting future studies. The lower concentration of 10 mM induced only a minor effect on fatty acid uptake and DPP4 activity. This might be associated with the important role of nutrient sensing as well, as glucose has been reported to have a sweetness detection threshold of 41.3 mM [55], thus a higher concentration than 10 mM might be needed to activate intestinal TAS1R3. Moreover, 10 mM of glucose is not high enough to induce trafficking of GLUT-2 to the apical side. This further supports that SGLT-1 does not unfold similar effects on fatty acid uptake, as also shown by the co-incubation experiments with phlorizin.

Incubation with the TAS1R3 agonists sucralose and NHDC in a similar sweetness range of glucose and sucrose did not affect fatty acid uptake to a similar extent as carbohydrates. In addition, the inhibitory effect of the disaccharide sucrose on fatty acid depended on the cleavage of the disaccharide into fructose and glucose. We conclude here that only the interplay of TAS1R3 and GLUT-2, but not TAS1R3 alone triggers the strong inhibition of fatty acid uptake by glucose. Such an interplay of glucose transporters and sweet taste receptor activation has been suggested in the regulation of energy intake and plasma GLP-1 by human studies before [56,57].

A well-known function of DPP4 is the cleavage of GLP-1 and GIP. Thus, we further investigated whether the incretin hormones GLP-1 and GIP are involved in the downstream reactions. The GLP-1 production in Caco-2 cells was under the limit of quantitation and also the genes for the GLP-1 receptor as well as GLP-1 were not expressed. In addition, stimulation of Caco-2 cells with 1 and 10 nM GLP-1 did not affect fatty acid uptake. It is possible that the basal DPP4 activity of Caco-2 cells influenced the GLP-1 concentrations, as the inhibition of the DPP4 activity, described already above, also led to a reduction of fatty acid uptake.

The second incretin hormone which should be considered is GIP. GIP has been recently discussed to be the more important incretin hormone in healthy humans [58,59]. Equally to GLP-1, Caco-2 cells were tested for their GIP production. In contrast to GLP-1, the genes encoding for the GIP receptor and GIP were expressed in differentiated Caco-2 cells, and GIP levels were increased by 14 % after incubation with 50 mM glucose compared to control. It is known that fasting and postprandial GIP levels are higher than those of GLP-1 [60,61], which might explain the detection of GIP and not GLP-1 in the cell model. Independent of the presence of GLP-1 and GIP in Caco-2 cells, treating the cells with GLP-1 and GIP in relevant concentrations of 1–10 nM did not reduce fatty acid uptake. Thus, a direct effect of DPP4 independently of the incretin hormones is assumed in Caco-2 cells, possibly via the fatty acid transporters. This conclusion is supported by an incretin receptor-independent mechanism for modulating the expression of genes important for lipid metabolism during prolonged DPP4 inhibition in mice [62].

Under the conditions tested, nutrient sensing had a stronger influence than the cellular energy status. This needs to be investigated in more detail in future studies.

In contrast to our assumption that glucose treatment will affect the expression of glucose transporters, only a minor effect after 90 min compared to control could be observed for the targets GLUT-2 and SGLT-1.

## 5 Conclusions

**Fig. 6:**
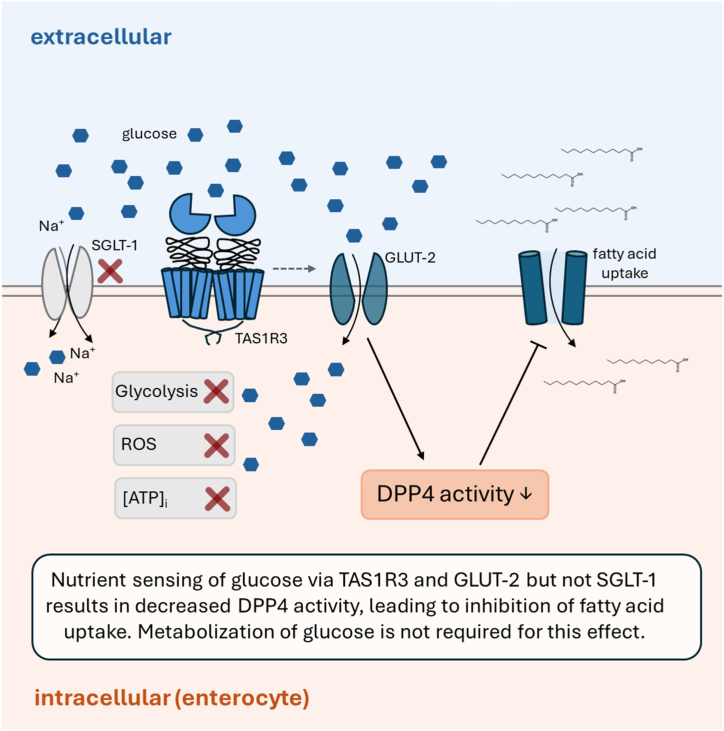
**Overview of the results and conclusion of the study**. Differentiated Caco-2 cells were used as a model for enterocytes to study fatty acid uptake and dipeptidyl peptidase-4 (DPP4) activity after treatment with the monosaccharide glucose. Glucose dose-dependently inhibited fatty acid uptake and decreased DPP4 activity. The pathway analysis showed that nutrient sensing via the sweet taste receptor subunit TAS1R3 and the glucose transporter 2 (GLUT-2) is required for the effect of glucose on fatty acid uptake and DPP4 activity while excluding (marked by the red cross) an effect of the sodium-coupled glucose transporter 1 (SGLT-1) and the requirement of metabolization of glucose (shown by the analyzed markers glycolysis, reactive oxygen species (ROS) and the intracellular ATP concentration ([ATP]_i_). (For interpretation of the references to color in this figure legend, the reader is referred to the Web version of this article.)

We demonstrate here for the first time that the presence of high amounts of glucose leads to a reduced DPP4 enzyme activity in Caco-2 cells via nutrient sensing of TAS1R3 and GLUT-2, which is associated with a reduced fatty acid uptake. An overview of the study results is provided in Fig. 6. Although the reduced DPP4 activity resulted in higher active GIP concentrations, GIP itself did not reduce fatty acid uptake. A direct connection of DPP4 and the fatty acid transporters is suggested by a decreased gene expression of FATP4, while at the same time, the gene encoding for CD36 was upregulated, possibly by the interaction with PPARγ. In conclusion, we identified DPP4 as a regulator of fatty acid uptake in Caco-2 enterocytes which mediates an inhibition of intestinal fatty acid uptake by glucose via an interplay of glucose transporters and the sweet taste receptor subunit TAS1R3. Remarkably, the nutrient sensing of carbohydrates via TAS1R3 and GLUT-2 was demonstrated to be a stronger regulatory factor of fatty acid uptake than an uncoupled ATP phosphorylation, which warrants further research.

Our findings are of relevance to understand how DPP4 unfolds plasma-lipid lowering effects in diabetic persons, as DPP4 is a target of common anti-diabetic treatments such as sitagliptin. Increased free fatty acid plasma concentrations have been associated with oxidative stress and pro-inflammatory effects [63] in healthy humans. In addition, elevated concentrations of free fatty acids lead to impaired insulin-mediated vasodilation [64], highlighting the importance of understanding the regulatory mechanisms of fatty acid absorption.

In addition, the strong effect of the interplay between the sweet taste receptor subunit TAS1R3 and glucose transporters, which even exceeds the effect of ATP phosphorylation, is of importance for understanding the metabolic effects of carbohydrates and sweeteners.

## 6 Limitation of the study

The results of the study show that TAS1R3 and GLUT-2 are required for mediating the effect of glucose on fatty acid uptake, however, the exact mechanism and downstream signaling warrants further research. Although the Caco-2 cell model is a well-established model for investigating intestinal absorption [29], it needs to be considered that it resembles only enterocytes and other cell types of the intestine are not considered. Therefore, further research with co-culture models or organoids would be needed to validate the results. This would be especially important to substantiate an incretin-receptor independent mechanism and investigate potential differences in the mechanistic action of GLP-1 and GIP. However, the mono-culture model of Caco-2 cells was here used as a starting point to address numerous metabolic pathways, resulting in the first mechanistic study that connects DPP4 activity with intestinal fatty acid uptake.

## Data availability statement

The data presented here are not available in a publicly available repository but are included in the article and supplementary material.

## CRediT authorship contribution statement

**Verena Preinfalk:** Writing – original draft, Visualization, Investigation, Formal analysis, Conceptualization. **Isabella Kimmeswenger:** Writing – review & editing, Investigation, Formal analysis. **Veronika Somoza:** Writing – review & editing, Resources. **Barbara Lieder:** Writing – original draft, Supervision, Project administration, Funding acquisition, Conceptualization.

## Declaration of competing interest

The authors declare the following financial interests/personal relationships which may be considered as potential competing interests:Barbara Lieder reports financial support was provided by Christian Doppler Laboratory for Taste Research, Christian Doppler Research Association. The Christian Doppler Laboratory for Taste Research is funded by the Austrian Federal Ministry of Labour and Economy, the 10.13039/100010132National Foundation for Research, Technology and Development, and the Symrise AG. If there are other authors, they declare that they have no known competing financial interests or personal relationships that could have appeared to influence the work reported in this paper.
