# Supplementary material for: Dipeptidyl-peptidase 4 (DPP4) mediates fatty acid uptake inhibition by glucose via TAS1R3 and GLUT-2 in Caco-2 enterocytes
Source: Heliyon. 2024 Apr 25;10(9):e30329. doi: 10.1016/j.heliyon.2024.e30329 (PMC11066672; doi:10.1016/j.heliyon.2024.e30329)
Supplement: Multimedia component 1 [file mmc1.docx]

**Supplementary figures and tables to**

**Dipeptidyl-peptidase 4 (DPP4) mediates fatty acid uptake inhibition by glucose via TAS1R3 and GLUT-2 in Caco-2 enterocytes**

Verena Preinfalk^1,2^, Isabella Kimmeswenger^2,3^, Veronika Somoza^3,4^, Barbara Lieder^1,3,5^

^1^ Christian Doppler Laboratory for Taste Research, Faculty of Chemistry, University of Vienna, Vienna, Austria

^2^ Vienna Doctoral School in Chemistry (DoSChem), University of Vienna, Vienna, Austria

^3^ Institute of Physiological Chemistry, Faculty of Chemistry, University of Vienna, Vienna, Austria

^4^ Leibniz Institute for Food Systems Biology at the Technical University of Munich, Freising, Germany

^5^ Institute of Clinical Nutrition, University of Hohenheim, Stuttgart, Germany

*Correspondence: Barbara Lieder, [Barbara.lieder@univie.ac.at](mailto:Barbara.lieder@univie.ac.at); Barbara.Lieder@uni-hohenheim.de

**Figure S1**

**
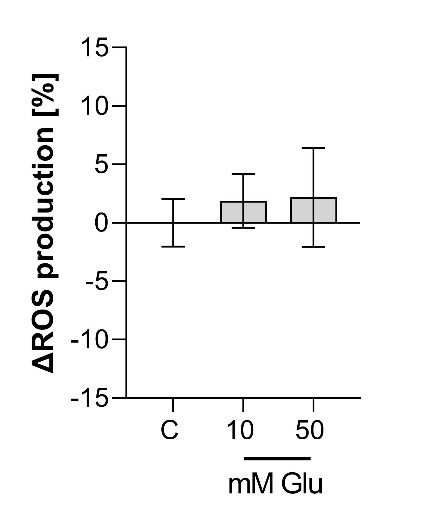
**

**Figure S1: ROS production in differentiated Caco-2 cells** after treatment with 10 or 50 mM glucose. Data are presented as Δ means compared to corresponding glucose with DMSO control ± SEM n = 3-4 (tr = 2). Significant differences were tested using One-Way ANOVA with Dunnett´s multiple comparisons test (p > 0.05).

**Figure S2**


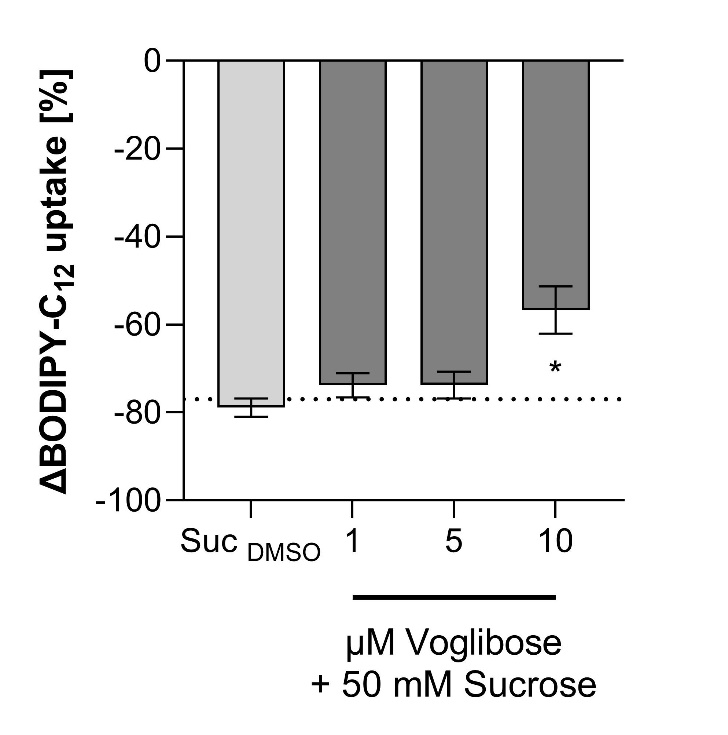


**Figure S2: BODIPY-C_12_ uptake by differentiated Caco-2 cells after pre-treatment with different concentrations of alpha-glucosidase inhibitor voglibose for 30 min, followed by sucrose for further 60 min.** Data are presented as Δ means compared to corresponding control ± SEM n = 3 (tr = 2). Significant differences were tested using one-way ANOVA with Tukey´s multiple comparisons test. Asterisk indicates statistically significant difference compared to control (50 mM sucrose + DMSO, *p < 0.05).

**Figure S3**


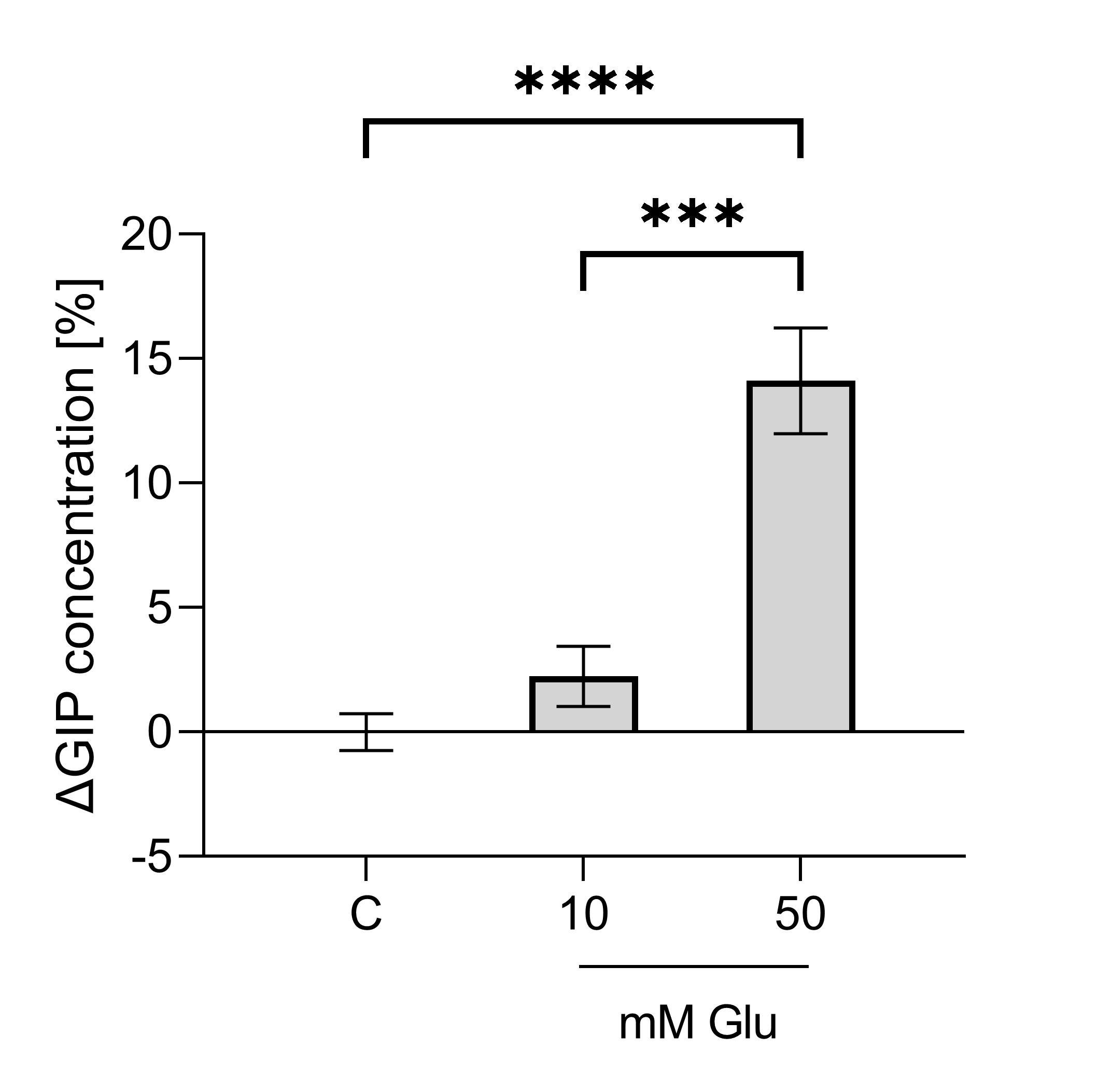


**Figure S3: GIP concentration [%] in differentiated Caco-2 cells after treatment with glucose.** Data are presented as Δ means compared to control ± SEM n = 3 (tr = 2). Significant differences were tested using One-Way ANOVA with Tukey´s multiple comparisons test. Asterisks indicate statistically significant differences compared to control (***p < 0.001, ****p < 0.0001).

**Table S1.**

| target | gene | Sequence (5´ 🡪 3´) | |
| --- | --- | --- | --- |
|  |  | **forward primer** | **reverse primer** |
| GAPDH | *GAPDH* | GTCTCCTCTGACTTCAACAGCG | ACCACCCTGTTGCTGTAGCCAA |
| CD36 | *CD36* | TGTAACCCAGGACGCTGAGG | GAAGGTTCGAAGATGGCACC |
| FATP2 | *SLC27A2* | TGGAACCACAGGTGCTACTC | ACCGAAGCAGTTCACCGATA |
| FATP4 | *SLC27A4* | GGCTGCCCTGGTGTACTATG | CCCACGATGTTTCCTGCTGA |
| PPARγ | *PPARG* | ACCAAAGTGCAATCAAAGTGGA | ATGAGGGAGTTGGAAGGCTCT |
| GLUT-2 | *SLC2A2* | CATGCTCTGGTCCCTGTCTGTATC | AACCCCATCAAGAGAGCTCCAACT |
| SGLT-1 | *SLC5A1* | CGCCTATCCAACCTTAGTGGTG | CGCTGTTGAAGATGGAGGTCAG |
| DPPIV | *DPP4* | AGTGGCGTGTTCAAGTGTGG | CAAGGTTGTCTTGTGGAGTTGG |
| GLP-1 | *GCG* | TGTCAGCGTAATATCTGTGAGGC | AGCAGGTGAAGAGAGAGCAAGC |
| GLP-1R | *GLP1R* | CAAATGCAGACTTGCCAAGTCCACG | CCAGCTGGACCTCATTGTTGACAAAG |
| GIP | *GIP* | TGGCAGTGGGACTAGGAGAG | GTTGAGGGCTGCTCACCTTA |
| GIP-R | *GIPR* | CACAATGTGAGAACCCAGAGAAGA | TGCAACCGCTCCAAGATGA |

**Table S1:** Sequences of the primers used in the present study
